# Folliculotropic Mycosis Fungoides Masquerading as Nodulocystic Acne: A Diagnostic Challenge

**DOI:** 10.7759/cureus.88442

**Published:** 2025-07-21

**Authors:** Santiago Leal, Melanie Villamizar, Paola Rojas, Xavier Rueda

**Affiliations:** 1 Dermatology, Instituto Nacional de Cancerología, Bogotá, COL; 2 Dermatology, Universidad Nacional de Colombia, Bogotá, COL

**Keywords:** acneiform eruptions, cutaneous t-cell lymphoma, folliculotropic mycosis fungoides, mimickers of acne, mycosis fungoides, nodulocystic acne, skin lymphoma, t-cell lymphoma

## Abstract

Mycosis fungoides (MF) is the most common form of cutaneous T-cell lymphoma, a type of skin cancer that involves malignant T lymphocytes infiltrating the skin. It typically presents as flat, red, scaly patches (erythematous plaques). A rare variant known as folliculotropic mycosis fungoides (FMF) differs in both its clinical behavior and histologic features. FMF is characterized by infiltration of hair follicles by malignant T cells and may present as deep, painful nodules that resemble common skin conditions such as nodulocystic acne, a severe form of acne with cysts and inflamed nodules. This clinical mimicry can lead to misdiagnosis, delayed treatment, and potentially worse outcomes.

Given its deceptive presentation and potential for progression, FMF should be considered in cases of persistent, treatment-resistant acneiform lesions, particularly in older patients. Here, we describe the case of an 85-year-old man with facial and truncal nodules initially treated as acne, who was ultimately diagnosed with FMF after histopathologic evaluation. This case underscores the importance of early biopsy and clinical suspicion to avoid misdiagnosis and ensure appropriate management.

## Introduction

Mycosis fungoides (MF) is a type of cutaneous T-cell lymphoma (CTCL) characterized by the infiltration of malignant T lymphocytes into the skin. While its classic presentation involves erythematous, scaly plaques, atypical variants can occur. One such variant is folliculotropic mycosis fungoides (FMF), in which follicular involvement can mimic various inflammatory dermatoses [1,2]. FMF may closely resemble common conditions such as nodulocystic acne, furunculosis, or even hidradenitis suppurativa, as all can present with deep, painful, inflammatory nodules [1,3,4]. In this case report, we describe a patient initially diagnosed with acne who was ultimately found to have FMF, highlighting the importance of comprehensive clinical evaluation and histopathological confirmation in cases with atypical or treatment-resistant presentations [2,4].

## Case presentation

**Figure 1:**
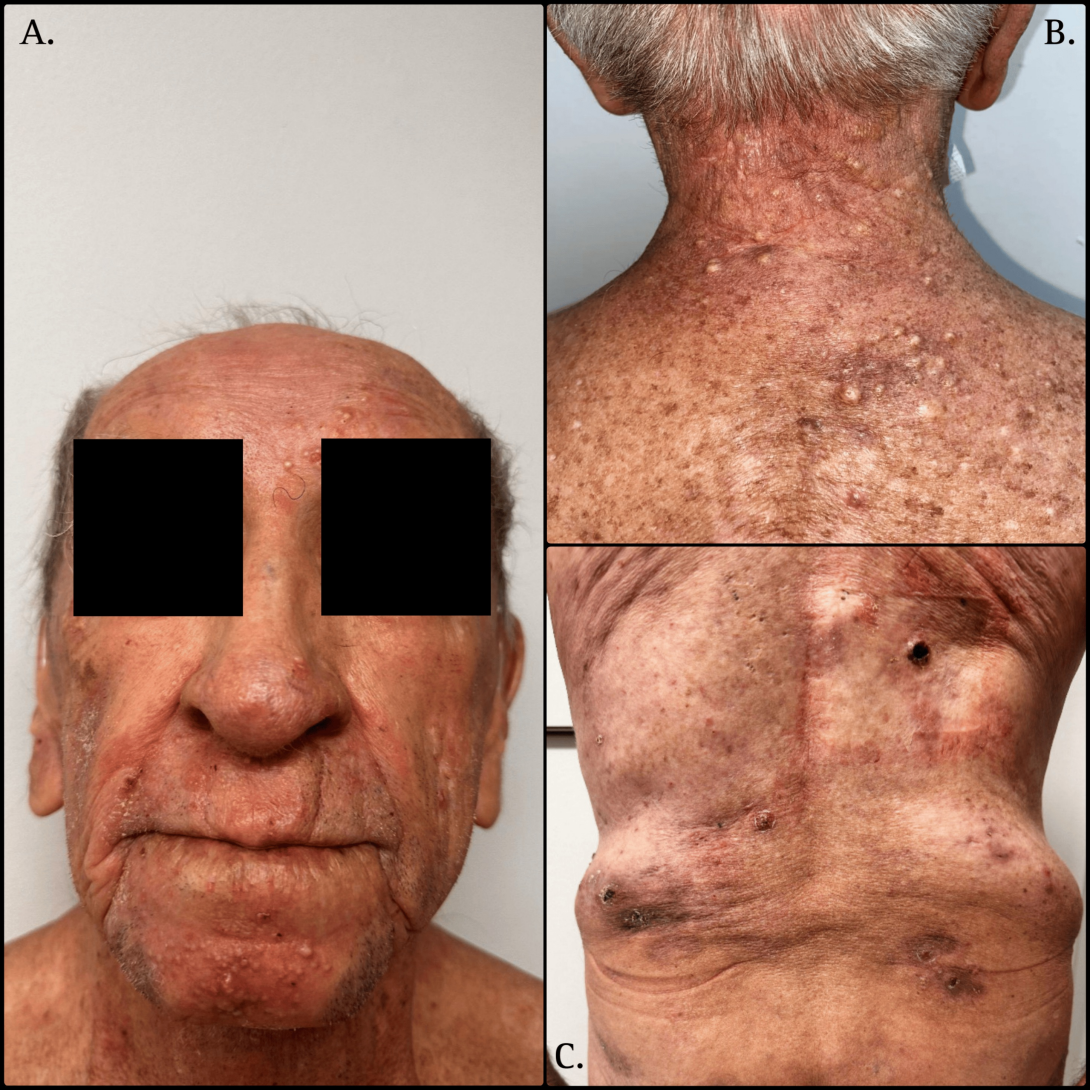
Clinical presentation. A. Infiltrated acneiform papules and nodules on the face of an 85-year-old male with folliculotropic mycosis fungoides; B. and C. infiltrated, ulcerated nodules primarily affecting sebaceous and follicle-rich areas such as the gluteal region and posterior thorax.

In 2019, an 85-year-old male developed pruritic, erythematous plaques on his arms, which gradually progressed over the following year to include infiltrated acneiform papules and nodules on the face, mimicking acneiform lesions. These were initially treated as nodulocystic acne with multiple topical therapies and systemic antibiotics, without clinical improvement. By 2020, the patient had also developed deep, painful, and occasionally ulcerated nodules on the posterior thorax and gluteal region, primarily affecting sebaceous and follicle-rich areas (Figure 1).

**Figure 2:**
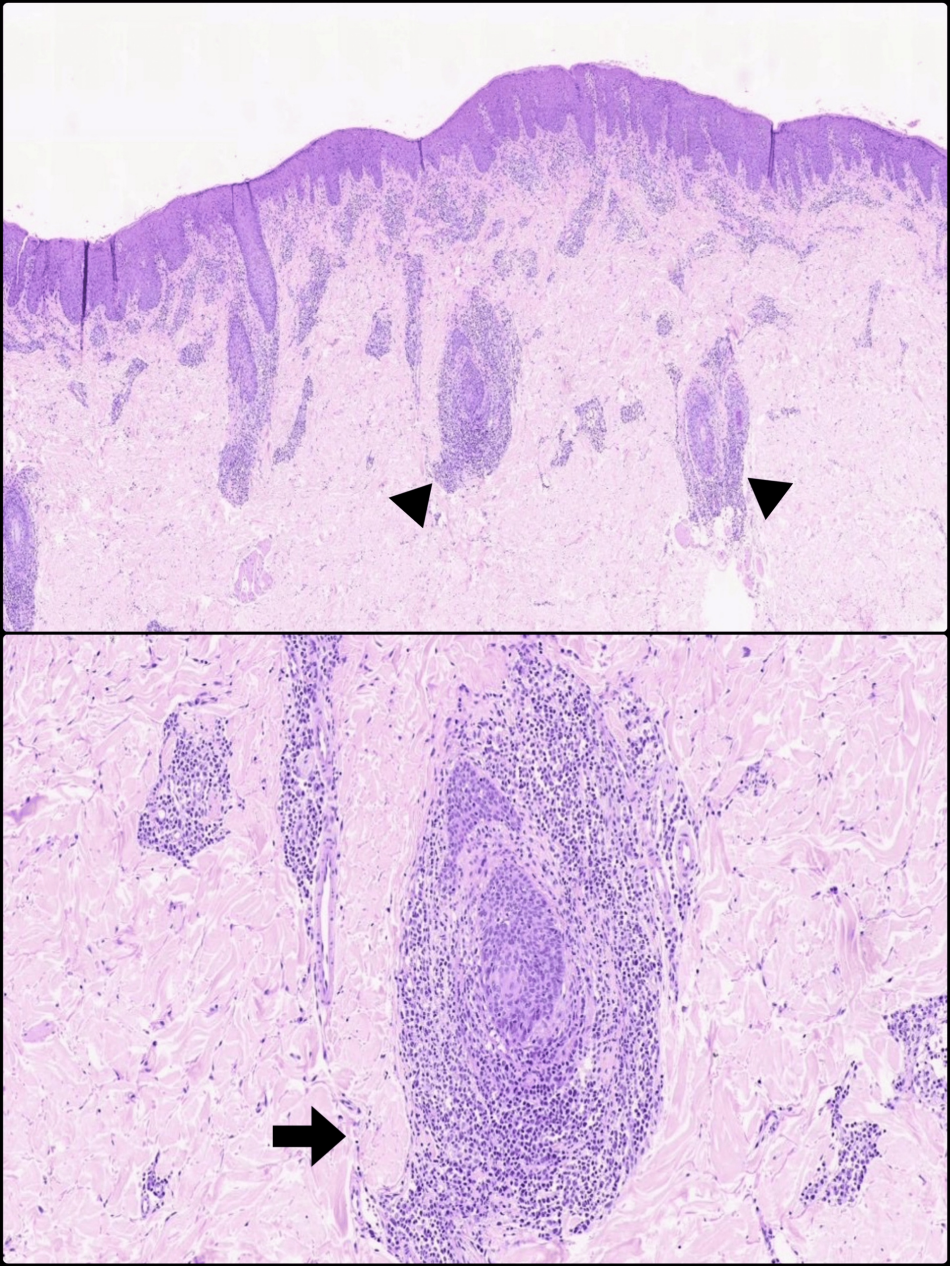
Histopathologic features of folliculotropic mycosis fungoides. Hematoxylin and eosin stain. (Top) Histopathology showing dense perifollicular lymphoid infiltrates involving hair follicles (triangles), consistent with folliculotropism. (Bottom) Higher magnification reveals mucinous degeneration of the follicular epithelium (arrow), a characteristic finding in this variant.

Due to the persistence of symptoms despite treatment, a skin biopsy was performed in 2021. Histopathological examination revealed an atypical lymphoid infiltrate with folliculotropism, meaning that the abnormal T cells showed a predilection for infiltrating hair follicles, a hallmark of folliculotropic mycosis fungoides. The tissue showed dense clusters of atypical lymphocytes surrounding and invading the hair follicles (perifollicular and folliculotropic aggregates), along with areas of mucinous degeneration (breakdown of follicular structures with mucin accumulation) and formation of follicular cysts. Additional findings included eosinophilic infiltrates (an influx of eosinophils, a type of white blood cell often involved in allergic or inflammatory processes) and focal epidermotropism, referring to scattered atypical lymphocytes migrating into the outer layer of the skin (epidermis) (Figure 2).

**Figure 3:**
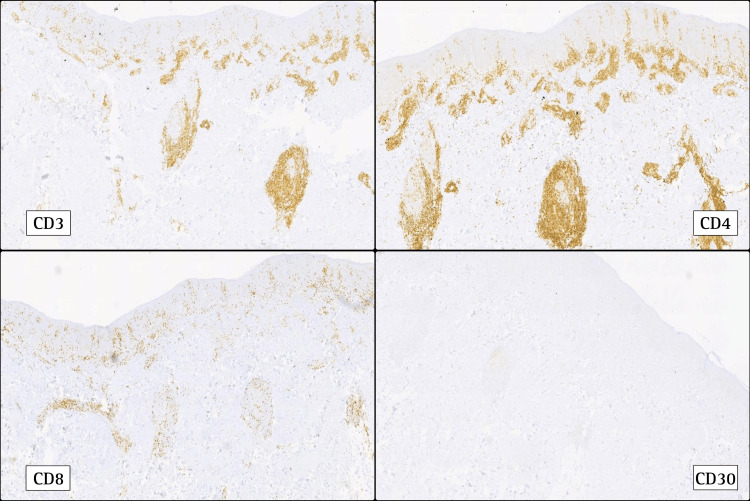
Immunohistochemical staining. CD3+, CD4+, CD8+, and CD30+ in 10% of atypical cells.

Immunohistochemical staining demonstrated a T-cell predominant-infiltrate with strong CD3 positivity and a CD4:CD8 ratio of approximately 2:1. Additionally, around 10% of the atypical lymphoid cells expressed CD30, supporting the diagnosis of folliculotropic mycosis fungoides (FMF) (Figure 3, panels A-D). The immunophenotype also showed reduced expression of CD7, a characteristic feature in MF, as well as mild downregulation of CD2. A few scattered CD20-positive B cells were also observed. These findings, combined with clinical features and histological folliculotropism, confirmed the diagnosis of FMF (Figure 3, panels A-D).

To further evaluate clonality and systemic involvement, flow cytometry (CMF) was performed on both bone marrow and peripheral blood samples. The bone marrow showed trilineage hematopoiesis with no evidence of lymphoid neoplasia. Flow cytometry revealed 8.23% of mature T lymphocytes expressing CD2, CD3, CD5, CD7, and CD45, with CD8 predominance and no aberrant marker loss. A subsequent peripheral blood flow cytometry (November 2022) confirmed a preserved CD4:CD8 ratio and absence of clonal T-cell populations or abnormal immunophenotypes.

Based on the TNMB staging system, the disease was classified as stage Ib, given the presence of multiple plaques and nodules involving more than 10% of the body surface area and the absence of extracutaneous involvement. Routine blood tests, including complete blood count and lactate dehydrogenase (LDH), were within normal limits. A PET-CT scan performed at the time showed no evidence of lymph node or visceral involvement.

After the histopathologic diagnosis of folliculotropic mycosis fungoides (FMF) was confirmed in 2021, the patient underwent multiple skin-directed treatments. These included high cumulative doses of UVA1 phototherapy (8,023 J/cm²), PUVA therapy (9,034 J/cm²), and topical corticosteroids. Due to limited response, he was subsequently started on interferon-alpha three times weekly from July 2023 to January 2024, followed by low-dose total skin electron beam therapy (TSEBT), using a modified Stanford technique (final dose 12 Gy), which concluded in March 2024. Although these therapies led to partial clinical improvement, his disease exhibited a refractory pattern, and new nodular lesions continued to develop.

In June 2024, systemic progression was confirmed by PET-CT, which revealed multiple hypermetabolic subcutaneous nodules (SUVmax up to 6.4), intraparotid lymphadenopathy (SUVmax 4.9-5.6), and a hypermetabolic lesion in the right lateral prostate (SUVmax 7.6). Brain MRI showed a left frontotemporal intraaxial lesion with perilesional edema and mass effect, suggestive of neoplastic involvement. A biopsy of a right costal subcutaneous lesion demonstrated transformation into peripheral T-cell lymphoma, not otherwise specified (PTCL-NOS), with focal GATA3 positivity and absence of p53 or CD30 expression.

Notably, although CD30 expression had been initially detected in approximately 10% of atypical lymphoid cells on the original skin biopsy, its loss in the transformed lesion limited the potential use of anti-CD30 targeted therapies such as brentuximab vedotin. Furthermore, focal GATA3 positivity, a marker associated with poor prognosis and chemotherapy resistance, supported the decision to avoid aggressive systemic regimens. Taking into account the patient’s advanced age, comorbidities, and suspected central nervous system (CNS) involvement, the multidisciplinary team opted for a palliative approach.

The patient is currently receiving liposomal doxorubicin (20 mg/m² on days 1 and 15, up to six cycles) and intralesional bleomycin for painful, ulcerated dorsal lesions. Supportive skin care includes daily topical corticosteroids and emollients. The treatment plan is focused on symptomatic relief and optimizing quality of life.

Informed written consent was obtained from the patient for the open-access publication of this case report.

## Discussion

Mycosis fungoides (MF) is the most common form of cutaneous T-cell lymphoma and typically presents in its early stages as pruritic, erythematous, and scaly patches or plaques. However, folliculotropic mycosis fungoides (FMF) represents a distinct clinicopathologic variant, characterized by a predilection for hair follicles and a markedly different clinical behavior. This variant often presents with deep-seated, infiltrated, and sometimes ulcerated nodules, primarily affecting sebaceous and follicle-rich areas such as the face, scalp, and trunk, features that may closely mimic nodulocystic acne, folliculitis, or other inflammatory dermatoses [1,2].

The pseudoinfectious and acneiform presentation of FMF can lead to diagnostic delays and mismanagement, particularly in elderly patients, where clinicians may initially suspect more common conditions. The lack of response to conventional acne treatments, including antibiotics and topical therapies, should prompt suspicion and warrant further investigation.

Given the overlapping clinical presentations, it is important to differentiate FMF from other acneiform or pseudoinfectious dermatoses. Nodulocystic acne, furunculosis, and hidradenitis suppurativa may all present with deep, painful, inflammatory nodules, particularly in sebaceous and follicle-rich areas. However, FMF more commonly affects elderly patients, tends to be more widespread, and typically lacks response to conventional therapies. Table 1 below provides a comparative summary of key clinical features among these conditions.

**Table 1:** Summarizes the distinguishing features between FMF and other clinically similar conditions. FMF: Folliculotropic mycosis fungoides

FEATURE	FMF	NODULOCYSTIC ACNE	FURUNCULOSIS	HIDRADENITIS SUPPURATIVA
Typical age group	Elderly adults	Adolescents/young adults	All ages	Young to middle-aged adults
Common locations	Face, scalp, trunk, gluteal area	Face, back, chest	Axillae, thighs, buttocks	Axillae, groin, inframammary folds
Lesion type	Infiltrated nodules, plaques	Papules, nodules, cysts	Painful pustules/nodules	Deep nodules, sinus tracts
Onset and evolution	Insidious, chronic	Gradual onset	Acute or recurrent	Chronic, relapsing-remitting
Response to antibiotics	Poor	Good	Usually good	Variable
Histology	Atypical lymphocytes with folliculotropism	Follicular rupture, neutrophils	Folliculitis with neutrophils	Follicular occlusion and inflammation
Clue to FMF	Poor response, age, histology	Teenage onset, facial predominance	Recurrent boils in specific areas	Double comedones, scarring sinus tracts

Histologically, FMF is defined by dense perifollicular and folliculotropic infiltrates of atypical lymphocytes, frequently accompanied by mucinous degeneration and follicular cystic changes. Immunohistochemistry typically reveals a T-cell phenotype with CD3 positivity, a CD4:CD8 ratio greater than 1, and variable loss of pan-T-cell markers such as CD2 or CD7. In our case, approximately 10% of atypical lymphocytes expressed CD30, a marker associated with more aggressive behavior and transformation risk. Although brentuximab vedotin, an anti-CD30 antibody-drug conjugate, has been used in some advanced cases of MF and PTCL, it was not pursued in our patient due to the subsequent loss of CD30 expression after transformation, as well as comorbidities and suspected CNS involvement.

FMF tends to follow a more aggressive and treatment-resistant course compared to classic MF. Management often requires a stepwise and multimodal approach, beginning with skin-directed therapies such as phototherapy (PUVA, UVA1) and topical corticosteroids, which are generally well tolerated and effective in early or limited diseases. In patients with partial or inadequate response, immunomodulatory agents like interferon-alpha may be added. Total skin electron beam therapy (TSEBT) is often reserved for refractory cutaneous disease due to its ability to induce remission across large body areas while minimizing systemic toxicity. In advanced or transformed cases, systemic chemotherapy or targeted therapies may be considered, depending on the immunophenotype and patient condition [2,4].

The present case highlights the importance of recognizing atypical presentations of cutaneous lymphomas and the need for early biopsy in cases of persistent or unusual nodular lesions. Dermatologists should maintain a high index of suspicion for lymphoproliferative disorders in patients with follicular, treatment-refractory dermatoses, especially when involving the face or upper trunk. Early and accurate diagnosis is critical, not only to initiate appropriate treatment but also to potentially improve prognosis, given the more aggressive nature of FMF compared to classic MF. Ultimately, awareness of FMF’s clinical variants remains essential for timely diagnosis and optimal patient outcomes.

## Conclusions

Folliculotropic mycosis fungoides is a rare variant of cutaneous T-cell lymphoma that can clinically mimic other dermatologic conditions, such as nodulocystic acne. Accurate diagnosis relies on skin biopsy and histopathological evaluation, underscoring the importance of thorough assessment in cases of treatment-resistant cutaneous lesions. Early treatment with phototherapy and topical agents may be effective in managing this condition, while long-term follow-up is essential to monitor recurrence and disease progression. This case highlights the acneiform and pseudoinfectious clinical presentation of FMF, which can mimic benign inflammatory dermatoses and delay appropriate diagnosis, and it also illustrates how FMF may initially respond to skin-directed therapies but can later evolve into more aggressive forms, requiring systemic treatment and palliative care. The persistence of follicular, treatment-resistant inflammatory lesions should raise clinical suspicion for cutaneous lymphoproliferative disorders, particularly in elderly patients.
